# 287. Procalcitonin and D-dimer levels at baseline, but not CRP, were informative of COVID-19 hospitalization outcomes

**DOI:** 10.1093/ofid/ofac492.365

**Published:** 2022-12-15

**Authors:** Trini Mathew, Julie George, Christine N Yost, Mustafa Deebajah, Paul C Johnson, James Huang, Christopher F Carpenter

**Affiliations:** Beaumont Hospital Royal Oak, Royal Oak, Michigan; Beaumont Hospital Royal Oak, Royal Oak, Michigan; Beaumont Hospital Royal Oak, Royal Oak, Michigan; Beaumont health system, Royal oak, Michigan; Beaumont Hospital, Royal Oak, Royal Oak, Michigan; Beaumont Hospital Royal Oak, Royal Oak, Michigan; Beaumont Hospital, Royal Oak, Royal Oak, Michigan

## Abstract

**Background:**

The WHO estimates 512 million cases of COVID-19 and 6.2 million deaths globally as of May 4^th^, 2022. In Michigan (MI), the first case was diagnosed March 10^th^, 2020. We describe here outcomes of COVID-19 patients cared for in a large tertiary hospital in 2020 spanning two surges based on baseline lab values for C-reactive protein (CRP), Procalcitonin (PC), and D-Dimer (DD).

**Methods:**

After IRB approval, adult patients diagnosed via PCR with COVID-19 during the two surges in 2020 and admitted to Beaumont Hospital, Royal Oak, an 1,131 beds tertiary care referral center in MI, were reviewed. Demographic, clinical and laboratory characteristics were obtained from the EMR. ICD-10 classification diagnoses were utilized to identify comorbidities, and patient BMIs were based on the admission values in the EMR. Outcomes were defined as death during current admission, transfer to nursing home or other facility, or discharge home. Using a tree-based model and the combined levels of the three labs we defined a hierarchy of four lab levels, each progressively having increased risk of death. We then analyzed the outcome for the four levels, adjusting for time period (surge), age, sex, race, BMI and comorbidities. Data was analyzed using SAS statistical software version 9.4 (SAS Institute).

**Results:**

A total of 2197 patients were identified from March through December 2020, of whom 1118 had CRP, PC and DD available at baseline. The mean age was 66.7 years (SD 16.1) for the cohort in first surge (March-June), and 66.4 (15.6) in the latter surge (July-December, Table1). More patients had a PC of >0.5 in the first surge (25.7%) than the second (13.2%). After adjusting for all other factors, the hierarchical lab levels are significantly associated with outcomes. Of note, baseline CRP value was not informative. Compared to the 2nd level (Table 2), the lowest level (PC < 0.1) has significantly lower odds of death [OR=0.37, 95% CI (0.19, 0.73)], while the highest level, (DD >1000 and PC ≥ 0.26) has significantly higher odds of death [OR=3.01, 95% CI (1.59, 5.72)].

**Figure:**
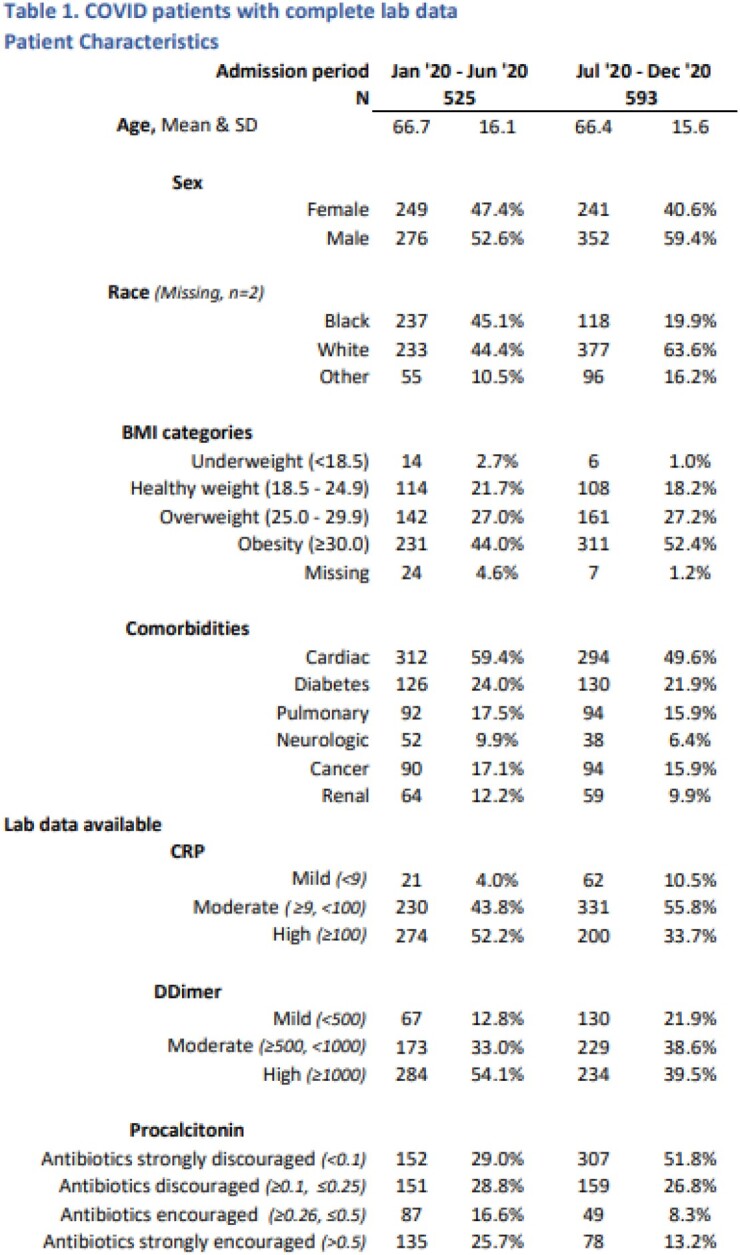

**Figure:**
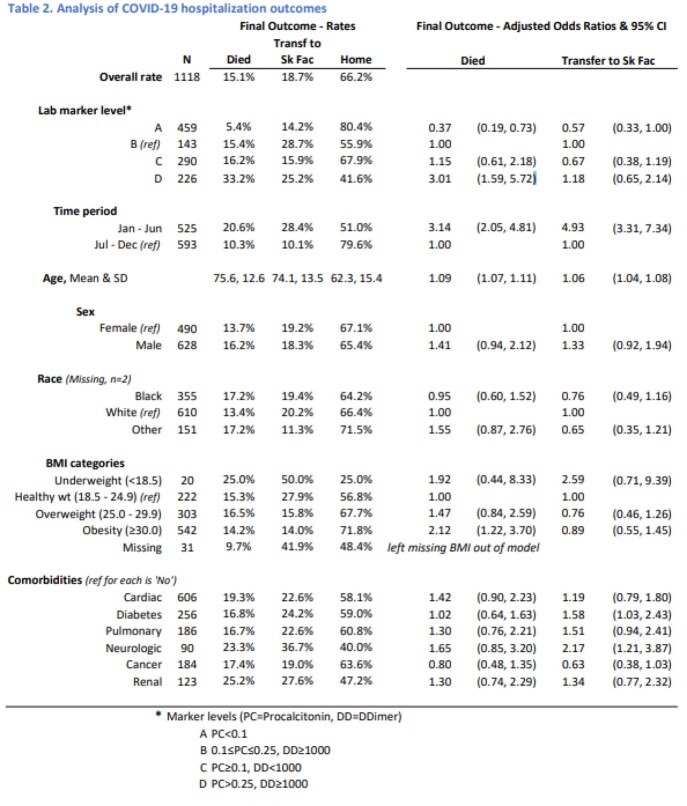

**Conclusion:**

Baseline PC and DD, but not CRP, were informative in determining risk of death and can assist clinicians determine possible outcomes during COVID-19 hospitalization.

**Disclosures:**

**Christopher F. Carpenter, MD, MHSA**, Atox Bio: Advisor/Consultant|GSK: Advisor/Consultant|Iterum Therapeutics: Advisor/Consultant|Takeda: Advisor/Consultant.

